# CRISPR/Cas9-Mediated Targeting of Susceptibility Factor *eIF4E*-Enhanced Resistance Against Potato Virus Y

**DOI:** 10.3389/fgene.2022.922019

**Published:** 2022-07-13

**Authors:** Azka Noureen, Muhammad Zuhaib Khan, Imran Amin, Tayyaba Zainab, Shahid Mansoor

**Affiliations:** ^1^ Agricultural Biotechnology Division, National Institute for Biotechnology and Genetic Engineering (NIBGE), Pakistan Institute of Engineering and Applied Sciences (PIEAS), Faisalabad, Pakistan; ^2^ University Institute of Biochemistry and Biotechnology (UIBB), Pir Mehr Ali Shah-Arid Agriculture University, Rawalpindi, Pakistan; ^3^ National Centre of Industrial Biotechnology (NCIB), Pir Mehr Ali Shah-Arid Agriculture University, Rawalpindi, Pakistan

**Keywords:** potato viruses, CRISPR-Cas9, *eIF4E*, PVY, DAS-ELISA, RT-PCR, *VPg*-EIF4E protein interaction

## Abstract

Potato (*Solanum tuberosum* L.) is an important staple food around the world, and potato virus Y (PVY) is a major constraint for potato production. The VPg protein of PVY interacts with the translation initiation factor *eIF4E* of the host that works as a susceptibility factor during infection. The interaction between eIF4E and VPg was disrupted by CRISPR/Cas9. The homozygous conserved region of *eIF4E* of the potato variety “Kruda” was mutated by CRISPR/Cas9. Tracking of insertion, deletion, and conversion events was performed by Sanger sequencing with ∼15% editing efficiency. Truncated and mutated eIF4E proteins were unable to interact with VPg, and the virus was not able to exploit the host machinery for replication and systemic spreading. Mutated *eIF4E* lines showed enhanced resistance to PVY^O^ strain. DAS-ELISA and RT-PCR were used for validation of the observed resistance. PVY resistance in tetraploid lines *via* CRISPR/Cas9 provides a route to develop novel resistant potato cultivars.

## 1 Introduction

Potato (*Solanum tuberosum* L*.*) belongs to the family Solanaceae and is a significant vegetative food crop grown on 19 million hectares worldwide. Asia is the largest potato-producing region (FAOSTAT, 2021; http://www.fao.org/faostat/en/#search/Potatoes). A potato reference genome was recently reassembled and annotated with updated gene annotation tools, which revealed more genetic diversity than can be monitored by molecular markers (van Lieshout et al., 2020). These markers help in identifying genetic variability, quantitative trait loci (QTLs), population structure, useful traits, and phenotypic variations (Sharma et al., 2018).

Sustainable potato production is greatly affected by biotic and abiotic constraints. Among biotic factors, RNA viruses significantly threaten crop yield. Potato virus Y (PVY), potato leaf roll virus (PLRV), potato virus A (PVA), potato mop-top virus (PMTV), potato virus X (PVX), and potato virus S (PVS) are the prominent potato viruses (Ali et al., 2002). Several non-recombinant (PVY^N^, PVY^O^, and PVY^C^) and recombinant strains (PVY, PVY^NW^, PVY^N:O^, and PVY^NTN^) of PVY have been reported. PVY has a global distribution and a broad host range. It is non-persistently transmitted by almost 50 different aphid species, with *Myzus persicae* as the main vector (Quenouille et al., 2013).

PVY encodes a replicase, a coat protein, a viral genome-linked protein (VPg), and a movement protein for systemic spread (Shukla et al., 1991; Gao et al., 2014; Wang et al., 2015). The ssRNA-potyviruses highjack translation initiation factors and related machinery of hosts for viral protein synthesis. The eIF4E protein plays a crucial role in the translation of the viral genome. Some natural allelic variations in this protein at the cap-binding site can confer virus resistance (Cavatorta et al., 2011). The mechanism of eIF4E-mediated recessive resistance against PVY has been explained (Moury et al., 2014a). These natural antiviral eIF4E variants affect the interaction of potyviruses with the host without disrupting the viability and cellular signaling. It has been previously reported that overexpression of the eIF4E-1-encoding pvr-1 gene from *Capsicum* provided resistance against *Tobacco etch virus* in *Solanum lycopersicum* (Kang et al., 2005). The potato eIF4E differs from pepper eIF4E allele *Pvr-1* in an amino acid substitution in the cap-binding domain which is essential for interacting with the RNA of PVY that ultimately disturbs the *VPg–*eIF4E complex formation (Kang et al., 2005; Kang et al., 2007). Furthermore, over-expressing the modified eIF4E and suppressing the susceptible allele transcript have provided potential resistance against PVY (Gutierrez Sanchez et al., 2020).

The recent advancements in targeted genome engineering via CRISPR/Cas have unprecedented potential to improve crops (Zhang et al., 2019; Dong et al., 2020; Liu et al., 2020; Zafar et al., 2021). Although genetic improvement in potato has limitations due to its genome complexity, it still presents a good opportunity to engineer useful traits. CRISPR/Cas9 is a widely adopted and sophisticated tool for crop genome engineering that specifically targets the desired gene(s) (Zafar et al., 2020a; Ibrahim et al., 2021). It can produce insertions, deletions, substitutions, or point mutations (SNPs) at a specified location in the target gene. Susceptibility genes provide potential targets for genome editing against viral, bacterial, and fungal pathogens as well as abiotic stresses (Zaidi et al., 2018; Zafar et al., 2020a). In this study, we used CRISPR/Cas9 to mutate the susceptibility gene *eIF4E* for engineering broad-spectrum resistance against PVY in an elite tetraploid potato cultivar ‘Kruda’. The mutated lines of the *eIF4E* gene showed enhanced resistance against PVY and can be used as a new genetic resource for the development of disease-resistant potato cultivars.

## 2 Results

### 2.1 Construct Assembly

**FIGURE 1:**
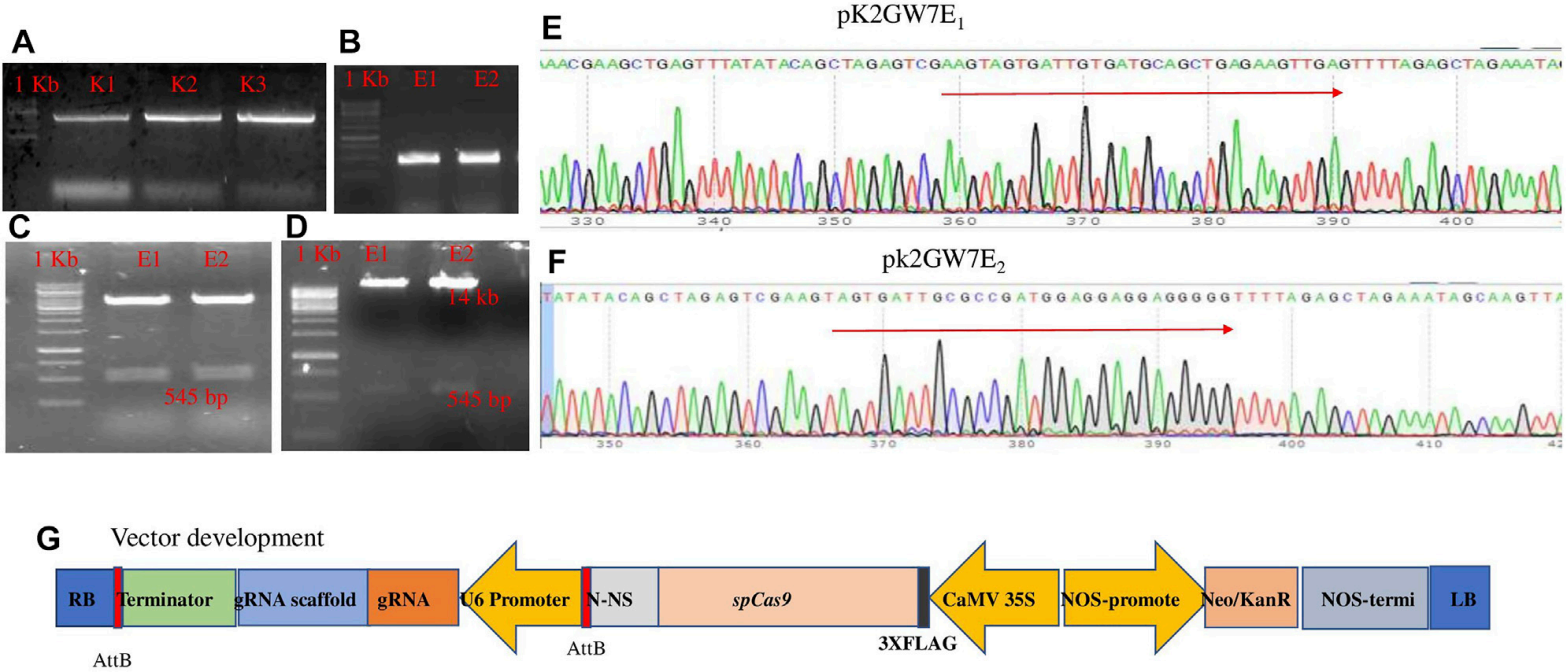
**(A)** Amplification of *eIF4E* gene and the 4.5-kb fragment; **(B)** amplification using the PCF/PCR primer set and the 556-bp fragment; **(C)** confirmation of gRNA in each construct by restriction analysis with the Bbs1 site in p. chimera vector; **(D)** the gRNA cassette confirmation in the Pk2GW7-*Cas9* vector (uncropped gel pictures with +ve and −ve controls are present in Supplementary Figures S4A–C); **(E)** pK2GW7E_1;_; **(F)** pk2GW7E_2_ through Sanger sequence confirmation of the gRNA cassette; **(G)** schematic diagram of the construct used for genome editing. All gRNAs targeting *eIF4E* were cloned in the same manner. The construct was expressing gRNA under the *At-U6* promoter. The plant codon-optimized *Cas9* was expressed under the 35S CaMV promoter.

The plant codon-optimized *Streptococcus pyogenes* Cas9 (*sp*Cas9) was expressed under the 35S promoter in the binary expression vector pK2GW7. Two constructs (PK2GW7-Cas9-E1 and PK2GW7-Cas9-E2) were developed to target the *eIF4E* gene (Table 1). The *eIF4E* gene was amplified by specific primers (Figure 1A), and gRNAs were designed on the conserved region of exon-1 (sequencing data in Supplementary Figure S1A). The PCR product confirmed the presence of the gRNA cassette (U6 *promoter*, gRNA, and gRNA scaffold) in the vector (Figure 1B). The 545-bp bands were eluted from a chimeric plasmid cloning vector and transferred to the expression vector pK2GW7 by *HindIII* restriction-ligation reactions (Figures 1C,D). The PCR results confirmed the presence of Cas9 (∼4.1 kb; Supplementary Figure S4A). The final constructs were confirmed by Sanger sequencing (Figures 1E,F). A complete diagrammatic illustration of the vector map is shown in Figure 1G.

**TABLE 1:** *eIF4E* gRNA sequences.

Gene	sgRNA	Sequence
*eIF4E* (gRNA1)	F	5′ATT​GTG​ATG​CAG​CTG​AGA​AGT​TGA 3′
	R	5′AAA​CTC​AAC​TTC​TCA​GCT​GCA​TCA 3′
*eIF4E* (gRNA2)	F	5′ATT​GCG​CCG​ATG​GAG​GAG​GAG​GGG 3′
	R	5′AAA​CCC​CCT​CCT​CCT​CCA​TCG​GCG 3′

### 2.2 Transgenic Plant Development

**FIGURE 2:**
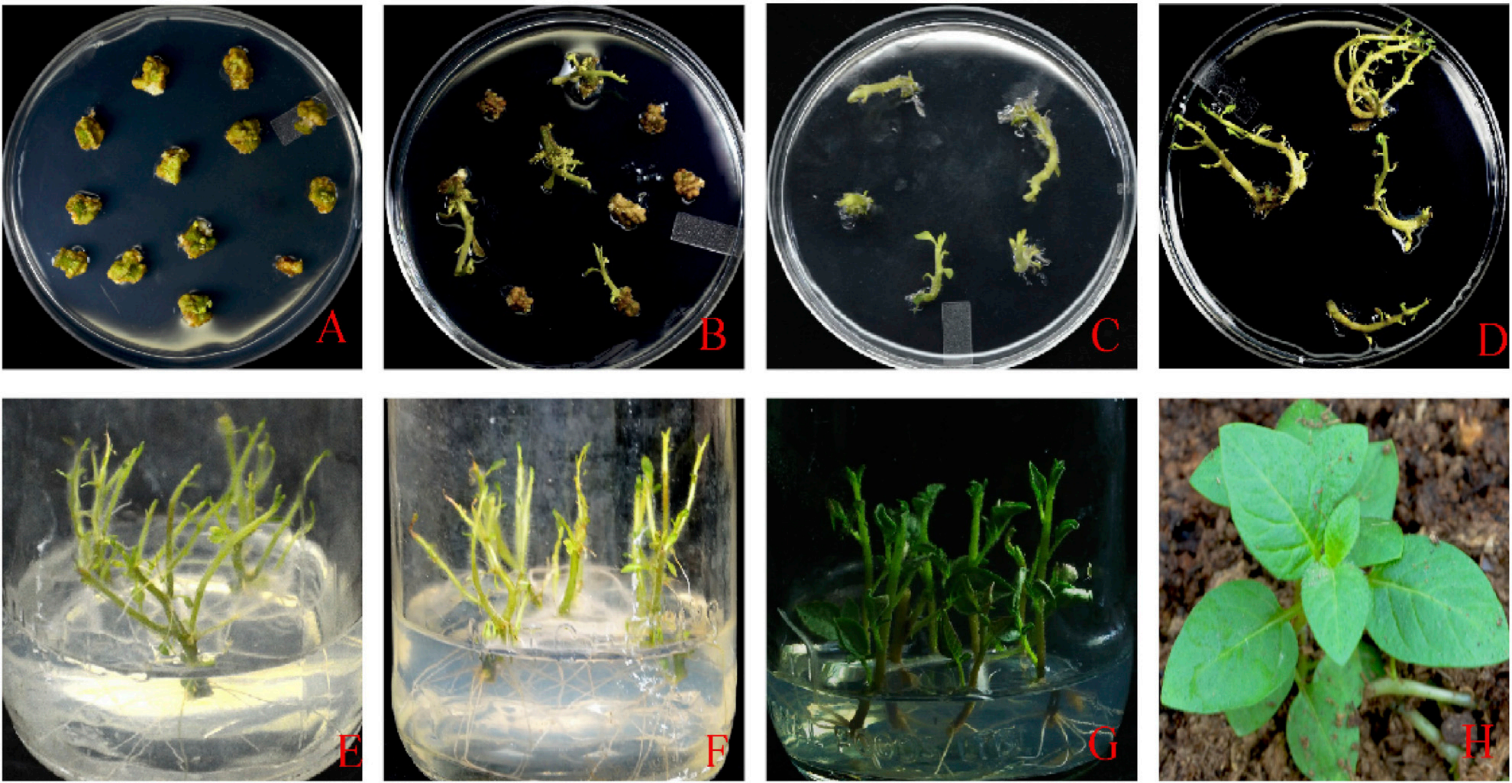
**(A)** Callus induction and development stage; **(B)** callus development and selection; **(C)** callus growth; **(D)** shooting stage; **(E–G)** shoot and root growth; **(H)** plants shifted to the glass house.

*Agrobacterium*-mediated transformation (GV3101 strain) generated 25 transgenic lines for gRNA1 and 15 lines for gRNA2 targeting the *eIF4E* gene. The mutated lines were confirmed by Sanger sequencing and multiplied before shifting to soil (Figure 2).

### 2.3 Editing Confirmation

The editing by Cas9 was tested by PCR amplification of the targeted regions of *eIF4E* and Sanger sequencing. Initially, PCR was performed to confirm the integration of Cas9 and the gRNA cassette into the plant genome and was followed by sequencing. We sequenced a total of 40 PCR-positive transgenic lines. In 25 of the 40 lines, *eIF4E* was targeted with gRNA1, and with gRNA2 in the other 15. The targeting efficiency of the CRISPR/Cas9 system in “Kruda” cultivar was around 15%. The targeted region of *eIF4E* was sequenced to determine the mutations. The purified PCR product was ligated into the pTZ57R/T vector by TA cloning, and 20 clones from each line were sequenced. The target regions were successfully mutated by insertion/deletion (Indels) and incorporation of single-nucleotide polymorphisms (SNPs). The gRNA1 targeted the most conserved region of the *eIF4E* gene near 5′-UTR. In the mutated lines K_E1.8, K_E1.9, K_E1.16, and K_E1.46, *eIF4E* was targeted with gRNA1, and the editing events were characterized by Sanger sequencing, which confirmed large deletions in one of the alleles of 25 bp, 15 bp, and 139 bp and insertion of 10 bp, 35 bp, and 42 bp in the K_E1.16 and K_E1.46 lines, respectively (shown in Table 2; Supplementary Figures S1A–D). We found that the SNPs in the coding regions in K_E1.46 generate stop codons that ultimately truncate the protein synthesis. The gRNA2 targeting *eIF4E* generated SNPs in K_E2.9 and K_E2.13 lines (Supplementary Figures S1E,F). In K_E2.9, a nucleotide with single base pair polymorphism and 3-bp deletions was observed, while in K_E2.13, three different events (a 1-bp addition and 3- and 1-bp deletions) were observed (Table 2, with gRNAs in red, PAM in purple, and editing events in green). These are synonymous mutations. We validated the resistance/tolerance capacity of these mutated alleles by ELISA and reverse transcription–polymerase chain reaction (RT-PCR).

**TABLE 2:** Editing events confirmation at allelic base through Sanger sequencing.

Line	Wild-type	Sequences (5′…….3′)	Editing event
Wild-type		ATGGCAGCAGCTGAAATGGAGAGAACGACGTCGTTTGATGCAGCTGAGAAGTTGAAGGCCGCCGATGGA	
E1.8	Allele 1	ATGGCAGCAGCTGAAATGGAGAGAACGACCTCGTTTGATGCAGCTGA-AAGTTGAAGGCCGACGATGGA	0
	Allele 2	ATGGCAGCAGCTGAAATGGAGAGAACGACCTCGTTTGATGCAGCTGA-AAGTTGA AGGCCGACGATGGA	0
	Allele 3	ATGGCAGCAGCTGAA-TGGAGAGAA ACGACCTCGTTTGATGCG/CGCTGA-AAAGTTGAAGGCCGACGAG	−1 bp Substitution −1 bp, +1 bp
	Allele 4	ATGGCAGCAGCTGAAATGGAGAGAACGACCTCCTTTGATGCAGCTGA-----AGTTGAAGGCCGACGATGGA	−1
E1.9	Allele 1	ATGGCAGCAGCTGAAATGGAGAGAACGACGTCGTTTGATGCAGCTGAGAAAG-GAAGGCCGACGATGGA	−1 bp
	Allele 2	ATGGCAGCAGCTGAAATGGAGAGAACGACGTCGTTTGATGCAGCTGAG--AGTTGAAGGCCGACGATGGA	−2 bp
	Allele 3	ATGGCAGCAGCTGAAATGGAGAGAACGACGTCGTTTGA---CAGCTGAGAAGTTGAAGGCCGACGATGGAG	−1 bp
	Allele 4	ATGGCAGCAGCTGAAATGGAGAGAACGACGTCGTTTGATGCAGCTGAGAAGTTGAAGGCCGACGATGGA	0 bp
E1.16	Allele 1	ATGGCCGCAGCTGAAATGGAGACGAA-GGAGCATATA------------------------------- ----------------GATGGAG	−25/+10 bp
	Allele 2	ATGG-AGAG--AACGACGAGGA---GCATATATAGGTGAGGGAAGAGAGCATATATGGAGACTTTCCAGATC	−15 bp/+14 bp
	Allele 3	ATGGAGAGAACGACGAGG-AGCATATATATAGGTGAGGGGAAGAGAGCATATATGGAGAGACTTTCCAA	−25 bp/+35 bp
	Allele 4	ATGGCAGCAGCTGAAATGGAGAGAACGACGTCGTTTGATGCAGCTGAGAAGTTGAAGGCCGCCGATGGA	0 bp
E1.46	Allele 1	ATGGAGAGAACGACG----- ---------------CCGCATATCT---------------- CATCCATTGGAGCATTCATGGACTTT	−139 bp/+42 bp
	Allele 2	ATGGCAGCAGCTGAAATGGAGAGAACGACGTCGTTTGATGCAGCTGAGAAGTTGAAGGCCGACGATGGA	0 bp
	Allele 3	ATGGCAGCAGCTGAA-TGGAGAGAACGACGTCGTTTGATGCAGCTGAGAAGTTGAAGGCCGCCGATGGAG	−1 bp (stop codon)
	Allele 4	ATGGCAGCAGCTGAA-TGGAGAGAACGACGTCGTTTGATGCAGCTGAGAAGTTGAAGGCCGCCGATGGA	-1 bp (stop codon)
Wild-type		GAACGACGTCGTTTGATGCAGCTGAGAAGTTGAAGGCCGCCGATGGAGGAGGAGGGGAGGTAGACGATG	
E2.9	Allele 1	GAACGACGTCGT-TGATGCAGCTGAGAAGTTGAAGGCCGCCGATGGAGGAGGAGGGGAGGTAGACGATG	−1 bp
	Allele 2	GAACGACGTCGTTTGATGCAGCTGAGAAGTTGAAGGCCGCCGATGCAGGAGGAGGAGAGGTAGACGATG	2 bp substitutions
	Allele 3	GAACGACGTCGTTTGATGCAGCTGAGAAGTTGAAGGCCGCCGATGG---AGGAGGGGAGGTAGACGATG	−3 bp
	Allele 4	GAACGACGTCGTTTGATGCAGCTGAGAAGTTGAAGGCCGCCGATGCAGGAGGAGGAGAGGTAGACGAT	2 bp substitutions
E2.13	Allele 1	GAACGACGTCGTTTGATGCAGCTGAGAAGTTGAAGGCCGCCGATGGAGGAGGAGGGGAGGTAGACGATG	0 bp
	Allele 2	GAACGACGTCGTTTGATGCAGCTGAGAAGTTGAAGGCCGCCGATGCAGGAGGAGGGGAGGTAGACGATG	1 bp substitutions
	Allele 3	GAACGACGTCGTTTGATGCAGCTGAGAAGTTGAAGGCCGCCGATG---GAGGAGGGGAGGTAGACGATG	−3 bp
	Allele 4	GAACGACGTCGTTTGATGCAGCTGAGAAGTTGAAGCGCCGATGCAGGAGGAGGGGAGGTAGACGATG	−2 bp

### 2.4 Phenotyping and DAS-ELISA

**FIGURE 3:**
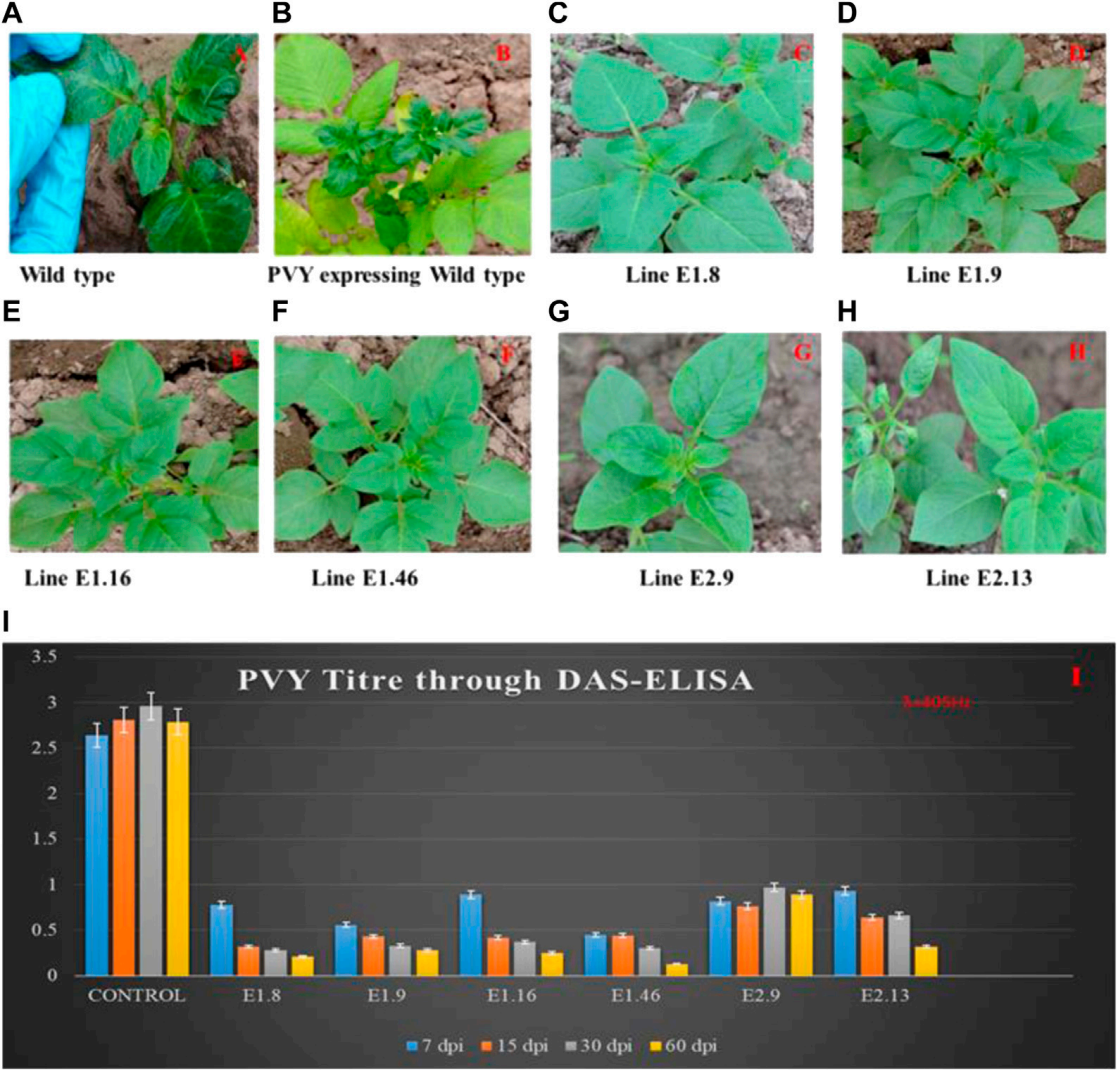
PVY Inoculation and symptoms appearance on control and mutated lines. **(A)** Inoculation of PVY into the control plant; **(B)** virus mosaic pattern appearance on the control plant at 45 dpi; lines **(C)** K_E1.8, **(D)** K_E1.9, **(E)** K_E1.16, and **(F)** K_E1.46 showing strong resistance, and lines **(G)** K_E2.9 and **(H)** K_E2.13 showing tolerance against PVY. **(I)** Determination of PVY titer using DAS-ELISA; The assay was performed to check the PVY titer at regular intervals of 7, 15, 30, and 60 dpi. The control lines showed high titer, while the mutated eIF4E lines K_E1.8, K_E1.9, K_E1.16, K_E1.46, K_E2.9, and K_E2.13 showed virus titer only after 60 days, which was lower than all others.

The resistance level of the mutated alleles was validated by ELISA and RT-PCR. Sixty-day mature mutated and wild-type lines were inoculated with PVY. Visual symptoms such as mosaic pattern on the leaves were observed on the wild-type plants (Figure 3). At 7, 15, 30, and 60 days post-infection (dpi), DAS-ELISA was performed. The analysis provided co-relating results with phenotypic observations: the wild-type lines exhibited common symptoms of PVY infection such as stunting, leaf mottling, crinkling, yellowing, and necrosis of leaves, while the mutated lines remained healthy without any visual symptoms of infection (Figure 3). The specificity and sensitivity of DAS-ELISA for PVY detection were confirmed by testing the extracts from PLRV-, PVX-, PVA-, PVS-, and PVM-infected potato samples along with negative controls. The negative results indicated the specificity and sensitivity of DAS-ELISA for PVY. The PVY titer was estimated through visual (yellow) observation and by ELISA-reading at 405 nm (Figure 3). Compared to the wild-type plants, all mutated lines showed a gradual decrease in virus titer and showed resistance at 60 dpi of PVY inoculation. The mutated lines K_E1.8, K_E1.9, K_E1.16, and K_E1.46 showed stronger resistance compared to K_E2.9 and K_E2.13 (Figure 3). Tubers were collected from PVY-resistant and PVY-tolerant lines, as shown in Supplementary Figures S2A,B. Phenotypically strong resistance was achieved by gRNA1 in lines K_E1.8, K_E1.9, K_E1.16, and K_E1.46, while tolerance was gained against PVY by gRNA2 in lines K_E2.9 and K_E2.13.

### 2.5 Relative Expression of *eIF4E and Cas9* in Mutated Lines

**FIGURE 4:**
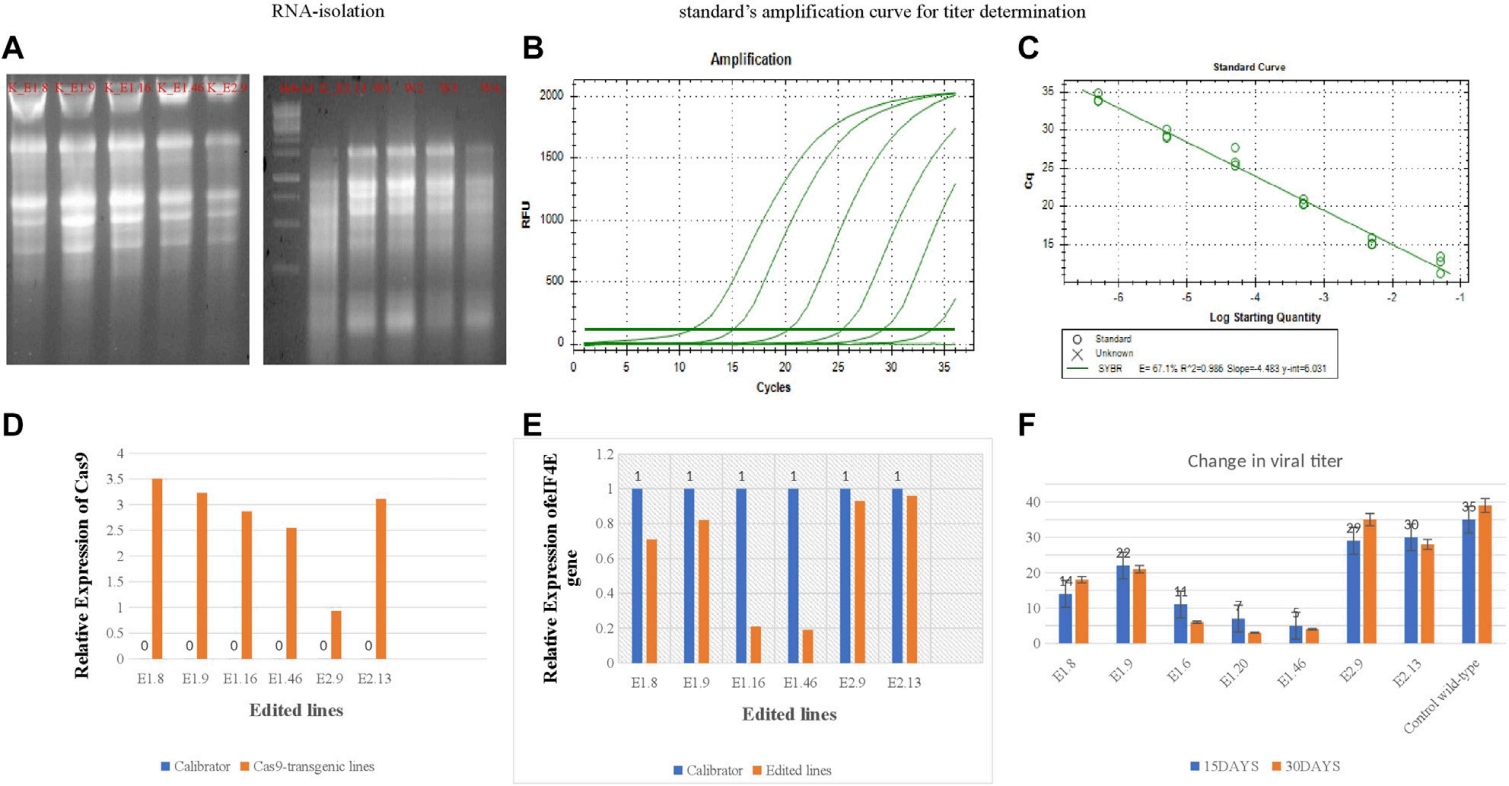
**(A)**; RNA-isolation. **(B,C)**: Stranded amplification curve for titre determination. **(C)**; The relative expression of *Cas9* in mutated lines (E1.8, E1.9, E1.16, E1.46, E2.9, E2.13) with wild-type control. **(D)**; The relative expression of *Cas9* gene in mutated lines (E1.8, E1.9, E1.16, E1.46, E2.9, E2.13) **(E)**; The relative expression of *eIF4E* gene in mutated lines (E1.8, E1.9, E1.16, E1.46, E2.9, E2.13) with wild-type control. **(F)**: The absolute quantification of viral accumulation in mutated lines and wild-type susceptible control line after regular interval of 15dpi, 30dpi.

Real-time PCR (RT-PCR) was performed to determine the functional expression of *eIF4E* and *Cas9* in tetraploid plants. The plants were treated with PVY and classified into the following: 1) non-inoculated wild-type Kruda as negative control; 2) PVY-inoculated wild-type Kruda as positive control; and 3) PVY-inoculated K_E1.8, K_E1.9, K_E1.16, K_E1.46, K_E2.9, and K_E2.13 *eIF4E* mutated lines. After 20 days of inoculation, symptoms appeared on the inoculated wild-type Kruda plants. Samples were collected at 20 and 30 dpi from PVY-resistant mutated and wild-type lines. The presence of virus in the samples was confirmed by DAS-ELISA. Total RNA was isolated, and cDNA was synthesized for the quantification of the relative expression of *eIF4E* and *Cas9* genes (Supplementary Figure S3A). To determine the functional expression of Cas9 in these mutated lines, the relative expression of Cas9 in mutated lines K_E1.8, K_E1.9, K_E1.16, K_E1.46, K_E2.9, and K_E2.13 was determined by using specific primers (shown in Table 3). The relative expression of *Cas9* gene is shown in Figure 4. The relative expression of *eIF4E* was higher in the wild-type control plant than in mutated lines, as determined by the 2^−ΔΔCT^ method (using primers shown in Table 3). The quantitative expression of *eIF4E* in mutated lines K_E1.8, K_E1.9, K_E1.16, K_E1.46, K_E2.9, and K_E2.13 was many folds lower than that in the wild-type (Figure 4), as indels caused the frameshift for the expression of *eFI4E*.

**TABLE 3:** Primers sequences used in RT-PCR.

Name	Sequence
St Actin1	St-AcF1: 5′GAT​GGC​AGA​CGG​AGA​GGA3′
	St-AcR1: 5′GAG​GAC​AGG​ATG​CTC​CTC3′
St Actin2	St-AcF2: 5′GTG​ACA​ATG​GAA​CTG​GAA​TGG​TCA​AGG​TAA3′
	St-AcR2: 5′GAC​CCA​TAC​CCA​CCA​TCA​CAC​CAG​TAT​GGC3′
*eIF4E* (RT-F&RT-R)	5′ ATG​GCA​GCA​GCT​GAA​ATG​GAG​AGA​ACG​AC3′
	5′ AGT​GAG​CTT​CCC​CAA​GCA​GTT​TGT​CGA​G3′
Cas9 (RT-F&RT-R)	5′ GGA​CTC​CCG​GAT​GAA​CAC​TA3′
	5′ TCG​CTT​TCC​AGC​TTA​GGG​TA3′
VPg (RT-vp1&2)	5′GAA​TTC​AAG​CCT​TGA​AGT​TTC​GCC​ATG​C3′
	5′TGC​GCC​CCA​GTG​AGT​GGA​TCA​ACG​AAT​T3′

### 2.6 Validation of Absolute Quantification Assay for Determination of PVY Titer in e*IF4E* Mutated Lines

The PVY pathosystem in ‘Kruda’ cultivar was validated by determining the viral load in the mutated lines and quantified by quantitative reverse transcription PCR (RT-qPCR). The optimal thermal cycling conditions were used to obtain the standard curve, with almost equal efficiency of amplification of the samples as shown in Supplementary Figure S3B. The mutated lines K_E1.8, K_E1.9, K_E1.16, K_E1.46, K_E2.9, and K_E2.13 of *eIF4E* and wild-type plants were inoculated with PVY. The copy number of PVY was determined by RT-qPCR, which indicated low or very low titer in mutated lines, compared to inoculated wild-type plants. The serial dilutions for RT-qPCR are shown in Table 4. The mutated lines E1.46 and E1.16 showed very low to zero virus accumulation and transmission at regular intervals. Interestingly, a decrease in the intensity of viral titer was observed in the E1.8 line at 45 dpi, compared to the wild-type plants (Figure 4). The confirmed resistant mutated lines were shifted from pots to soil to collect the tubers.

**TABLE 4:** Serial dilution of plasmid.

S. no.	Standard	Concentration (ng/µl)	Concentration in scientific notation	Copy number
1	S2	0.05	5.000E-02	2.3.E+08
2	S3	0.005	5.000E-03	2.3.E+07
3	S4	0.0005	5.000E-04	2.3.E+06
4	S5	0.00005	5.000E-05	2.3.E+05
5	S6	0.000005	5.000E-06	2.3.E+04
6	S7	0.0000005	5.000E-07	2.3.E+03

## 3 Discussion

Plant viruses recruit the host’s cellular machinery to translate their viral genome. It has been reported that positive-sense RNA viruses are facilitated by the eIF4E protein, which enables translation initiation of the viral genome and systemic spreading of the virus (Sanfaçon, 2015). The interaction between the eIF4E protein and VPg is required for the onset of viral genome protein synthesis and systemic dissemination of disease (Charron et al., 2008; Moury et al., 2014b).

Resistance can be established through mutations in the viral VPg gene. Plum pox virus strain C (PPV-C) is infectious to *Nicotiana* but non-infectious to *Arabidopsis* and *Chenopodium* species. PPV-C and PPV-D chimeric clones with VPg gene mutations are unable to interact with the eIF4E protein. These VPg gene changes have resulted in host-specific incompatibility, which leads to non-host resistance (Calvo et al., 2014). Introgression and overexpression of resistance genes (R-genes), alteration of susceptibility genes (S-genes), and host-derived resistance have all been identified as key strategies for inducing resistance in potato (Ellis et al., 2020).

**FIGURE 5:**
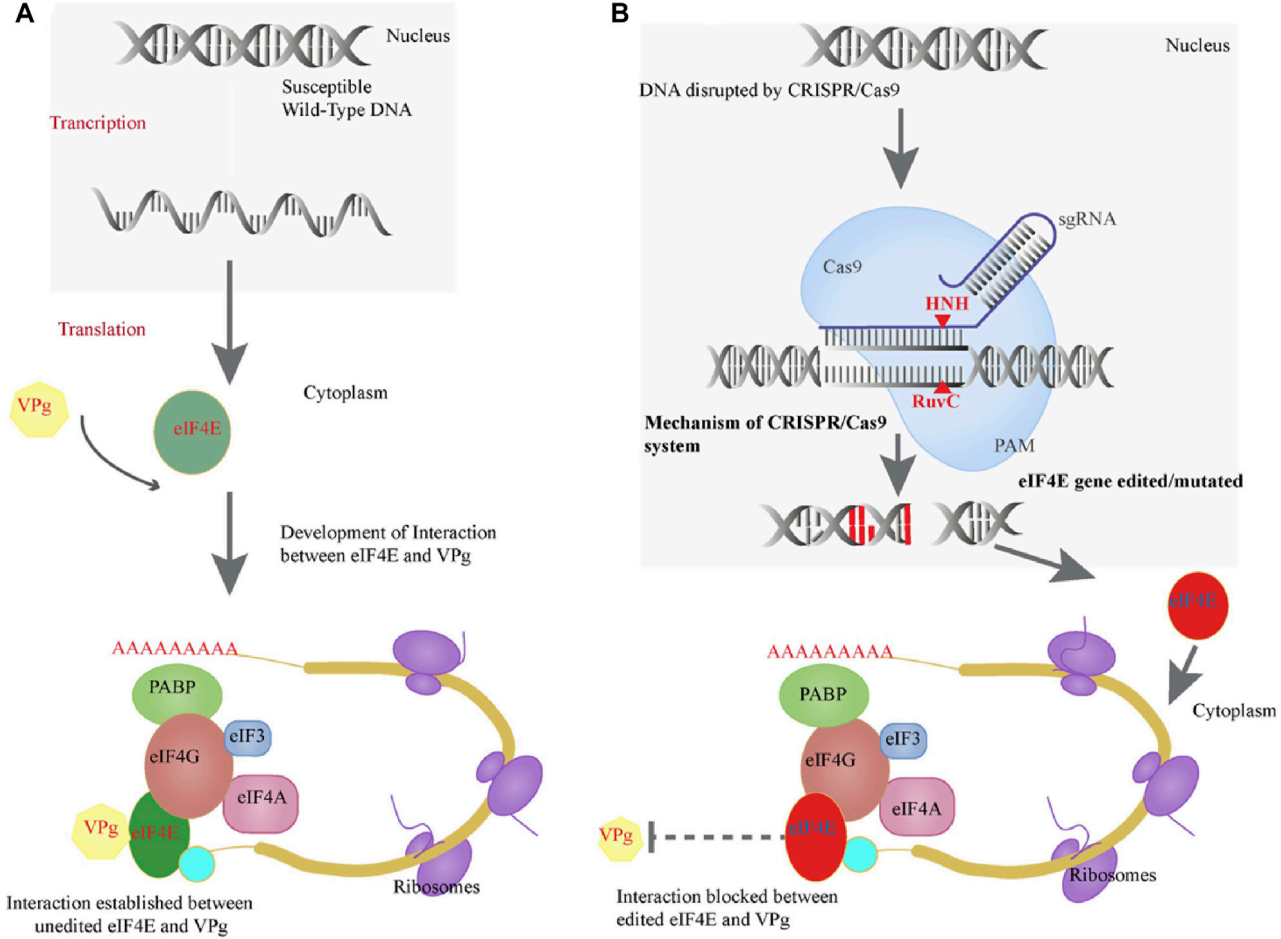
**(A)** Positive interaction of eIF4E protein of potato and VPg of PVY. **(B)** Genome editing efficiency of CRISPR/Cas and how it mutates the *eIF4E* and blocks the VPg–eIF4E interaction.

This is the first study to use CRISPR/Cas9 to mutate the *eIF4E* susceptibility gene in tetraploid potato cv. Kruda to generate resistance to PVY. Interaction was disrupted between VPg and eIF4E by the application of CRISPR/Cas9 that mutated *eIF4E* in tetraploid potato genome (Figures 5A,B). Previously, the CRISPR/Cas9 technology was used to inhibit the function of the recessive *eIF4E* gene, resulting in viral resistance in cucumber (*Cucumis sativus* L.) (Chandrasekaran et al., 2016). It has been reported that *Eva1*, a variant of *eIF4E-1*, provides resistance to PVY in *S. tuberosum*, *S. chacoense*, and *S. demissum.* Because Eva1 is unable to interact with VPg, which is required for pathogenesis to begin, intragenic potato cultivars with Eva1 are resistant to PVY (Duan et al., 2012). In the yeast two-hybrid system, the new variation Eva1 failed to interact with VPg (Duan et al., 2012). We mutated the *eIF4E* gene with CRISPR/Cas9 such that it could no longer interact with the VPg of PVY, resulting in PVY resistance (Figures 5A,B).

Using CRISPR/Cas9 followed by NHEJ-repair, we obtained several editing events in “Kruda” lines that resulted in insertions, deletions, point mutations, and SNPs. The NHEJ re-ligation is prone to errors, increasing the likelihood of insertions and deletions (indels) (Song and Stieger, 2017). NHEJ is the most common repair process in somatic cells. It is constitutively active throughout the cell cycle and is particularly efficient at fixing DSBs due to the efficiency of repair proteins ku70 and ku80. The CRISPR/Cas9 approach followed by NHJE-repair has been widely utilized to generate resistance in agricultural plants, such as in rice against bacterial blight, in cucumber against cucumber vein yellowing virus, and in tomato against powdery mildew (Chandrasekaran et al., 2016; Nekrasov et al., 2017; Shibata et al., 2018; Zafar et al., 2020b).

Editing all alleles in a polyploid genome simultaneously is complex and challenging. We exploited the *Arabidopsis* U6 promoter for expressing the CRISPR-array and obtained 15% mutation efficiency in targeting conserved tetraploid, homozygous regions of *eIF4E* homologs. The homozygous and hemizygous mutations in all alleles were confirmed through Sanger sequencing in the 6 mutated lines. However, we did not find any SNPs and somaclonal variations in tetraploid wild-type lines. Therefore, these mutated lines were generated through the potential activity of Cas9 endonuclease. We targeted the most conserved coding regions of *eIF4E* near the start of exon-1 that ultimately altered the open reading frame, leading to generation of truncated eIF4E proteins and slowing down of the onset of PVY disease. We also obtained some SNPs in potato that were synonymous mutations that had no effect on the protein’s open reading frame in lines E2.9 and E2.18; these lines showed minor resistance to PVY. Non-synonymous SNPs in the watermelon gene *eIF4E* have resulted in resistance to Zucchini yellow mosaic virus (Ling et al., 2009).

Previously, Andersson et al. (2018) exploited CRISPR/Cas9 to target the potato granule-bound starch synthase (*StGBSS*) gene. The mutagenesis frequency was 25%, while up to 9% indels were detected. Moreover, the authors reported a frequency of 2–3% mutations in all four alleles (Andersson et al., 2018). Furthermore, overexpression of the variant Potato4E: pvr1_2_ resistance allele resulted in resistance against PVY. This variant of the *eIF4E* gene differed from the susceptible allele by three point mutations and caused downregulation in endogenous susceptible *eIF4E.* No adverse effects were observed in plant growth (Cavatorta et al., 2011).

The adverse physiological effects of PVY^o^ were examined on the mutated lines. The mutated lines provided resistance against all tested PVY strains. We measured the virus titer by DAS-ELISA at regular intervals. The phenotypic data revealed that control plants exhibited the typical symptoms of PVY infection, whereas mutated lines K_E1.8, K_E1.9, K_E1.16, K_E1.46, K_E2.9, and K_E2.13 of *eIF4E* showed resistance at 10, 15, 25, 30, and 60 dpi. Furthermore, the virus accumulation in leaves was analyzed by RT-qPCR. Sensitive detection by RT-qPCR for addressing the viral load in mutated and control lines indicated high titers in the control plants compared to mutated lines. Previously, resistance against three strains of PVY (PVY^O^, PVY^N:O^, and PVY^NTN^) was developed by modifying *eIF4E* in the two potato varieties Atlantic and Russet Noekotah (Arcibal et al., 2016).

In tetraploids, genome heterogeneity, haplotype differences, and copy number variations (CNVs) may play a role in differential gene expression during biotic and abiotic stresses (Pham et al., 2017). The CRISPR/Cas9 constructs resulted in diverse allelic mutations in the *eIF4E* gene paralogs, and while these editing events provided resistance or tolerance against the devastating PVY, the physiology of the plants remained normal. This study is an experimental proof that the CRISPR/Cas9 system can be established in tetraploid potato. However, more studies are needed for developing Cas9-free lines against all strains of PVY. Furthermore, multiplex genome editing can be exploited for simultaneous targeting of multiple regions in *eIF4E* and other interacting isoforms, for developing broader resistance.

Conceivably, advanced bio-informatics tools could be exploited for deciphering the mechanism of protein–protein interaction of local cultivars and VPg of PVY strains. The specific amino acids that are involved in these interactions may be changed by using the variants of CRISPR/Cas9 such as cytidine base editing (CBE), adenine base editing (Zong and Gao, 2019), or prime editing (Wang et al., 2021). Thus, there is considerable scope to move forward from the proof-of-concept reported here, to verifying the findings with more varieties and additional editing methods.

## 4 Conclusion and Prospects

Clonal propagation of potato crop by tubers increases the possibility of virus accumulation in tubers. The CRISPR/Cas9 technology was used to create resistance to PVY, and resistant plants produced virus-free tubers. The CRISPR/Cas9 system-generated indels and SNPs in the potato *eIF4E* gene conferred resistance against a lethal strain of PVY. Therefore, CRISPR/Cas9 may be used to achieve targeted and beneficial varietal development. The establishment of broad-spectrum resistance against PVY could be a sustainable approach to fulfil the food demands of the growing population. However, the establishment of a transgene-free genome editing protocol—like the one using ribonucleoproteins (RNPs)—in potato is required for the development of globally accepted potato varieties, as the tetraploid nature of potato makes it challenging to recover a Cas9-free mutated line.

## 5 Materials and Methods

### 5.1 Plant Material

The tetraploid Kruda cultivar of potato was selected for establishing PVY resistance. Control plants were obtained from the Gene Transformation Lab (National Institute for Biotechnology and Genetic Engineering (NIBGE), Faisalabad, Pakistan). Two days prior to transformation, the internodal stem cuttings from control plants were placed on MS medium (Murashige and Skoog, 1962) in a growth chamber with a photoperiod of 16 h and a temperature of 22°C (±1°C). DAS-ELISA (Andersen and Johansen, 1998) was performed against single-stranded RNA (ssRNA) viruses for confirmation of virus-free parental lines.

### 5.2 gRNA Designing and Construct Development

In this study, *Solanum tuberosum* cv. Kruda plants were used. To find out the exact sequence of the susceptibility gene *eIF4E*, primers were designed on the NCBI-available sequence (NM_001288431) to amplify the coding region of the gene (Table 5). CTAB and Trizole (Invitrogen, United States) were used to extract genomic DNA (Doyle, 1990) and RNA, respectively, for the amplification of the coding region. PCR products of 635 bp for the coding sequence of *eIF4E* was amplified by using specific primers (Table 5, P1) according to the manufacturer’s standard PCR conditions (Catalog # K1082).

**TABLE 5:** Primers sequences.

Primers	Name	Sequence
P1	Eif4-F	5′ATG​GCA​GCA​GCT​GAA​ATG​GAG​AGA​ACG​AC3′
	Eif4-R	5′CTA​TAC​GGT​GTA​ACG​ATT​CTT​GGC​ACT​TCT​G3′
P2	PCF	5′TCC​AAA​GCT​TCC​TAG​GCT​TTT​TTT​CTT​C 3′
	PCR	5′CCA​GAA​GCT​TCT​AGG​TAA​TGC​CAA​CTT​T 3′
P3	CasD F	5′GCA​AGA​AAT​TCA​AGG​TGC​TGG​GCA​ACA3′
	CasD R	5′ACT​CTT​CCA​GTC​TGT​GGA​AGA​AGC​TGT3′
P4	PVYVpg-F	5′GTG​TCT​CAT​CAA​GGG​AAA​AAT​AAA​TCC3′
	PVYvpg-R	5′AAG​CCT​CTC​ATG​AGC​GAT​TTA​GCT​TCA​T3′
P5	EF1	5′ATG​GCA​GCA​GCT​GAA​ATG​GAG​AGA​ACG​AC3′
	ER1	5′ AGT​GAG​CTT​CCC​CAA​GCA​GTT​TGT​CGA​G3′
P6	KF	5′GCA​CGA​GGA​AGC​GGT​CAG​CCC​ATT​CGC​C3′
	KR	5′AGA​CCG​ACC​TGT​CCG​GTG​CCC​TGA​ATG​AAC3′

This coding sequence was cloned into Ptz57R/T (Catalog# K1213, Thermo Fisher Scientific, United States) for sequencing. The 20-bp gRNAs were designed on the conserved regions of exon1 of the *eIF4E* gene. All gRNAs were designed manually and screened for potential off-targets using the online Cas-OFFinder tool (http://www.rgenome.net/cas-offinder/). The gRNAs were cloned into the BbsI site of the p. chimera vector under the *A. thaliana-U6* promoter. The Pk2GW7-Cas9 construct was obtained from the Laboratory for Genome Engineering and Synthetic Biology, Centre for Desert Agriculture, KAUST, Saudi Arabia. The binary PK2GW7-Cas9 vector was designed with a unique *Hin*d III site, and the gRNA cassette was inserted into the Pk2GW7-SpCas9 vector using *Hin*d III restriction and ligation methods. Plant codon-optimized *sp*Cas9 (approximately 4.1 kb) was amplified by using high-fidelity polymerase (M0530S) following the manufacturer’s instructions, and further confirmation of Cas9 was performed using the diagnostic primer for Cas9 (Table 5).

### 5.3 Potato Transformation and Growth Conditions

The Kruda internode cuttings were placed on MS medium for 2 days prior to transformation by *Agrobacterium-*mediated transformation (GV^3101^ strain) harboring constructs. For each gRNA, approximately 100 cuttings were transformed. Each construct was grown independently with rifampicin and spectinomycin antibiotic selection. Optical density (O.D_600_) of the bacterial liquid culture was measured, and 0.6 O.D. was used for the transformation of the internodes. The liquid culture was pelleted down with 4430 g for 5 min and washed three times with liquid MS medium, and final O.D. was measured for transformation. A time duration of 20–25 min was used for the incubation of internodes in the liquid MS medium with the *Agrobacterium* construct. The cuttings were placed in a dark room overnight at 25–28°C for potato transformation. After overnight incubation, the transformed internodal cuttings were washed with timentin and then placed on kanamycin medium (50 mg/L) for selection. The transformed lines were transferred to callus induction medium (CIM) for callus induction. The cuttings were transferred continuously on a weekly basis for up to 40 days. The surviving calli were then transferred to regeneration medium, and regenerated plantlets were transferred to shooting medium followed by rooting medium with variable amounts of plant growth hormones. Before transferring to soil (clay and sand), the mutated lines were multiplied in a growth chamber, and three clonal replicates of each mutated line were transferred to sand and kept in a greenhouse under controlled conditions (25°C (±1°C) temperature, light period for 16 h, and with insect/pest proof). After 2 weeks of hardening, the lines were shifted into large pots for tuber development and phenotypic assays.

### 5.4 Confirmation of Transgenic and Mutated Lines

The transgenic lines were confirmed for the presence of Cas9 and the gRNA cassette. DNA was extracted from the mutated and control lines using the CTAB method. P. chimera forward (PCF) and reverse (PCR) primers (Table 5), Cas9 primers, and pNeomycin phosphotransferase II (*NptII*) gene (responsible for kanamycin resistance) primers (Table 5) were used for the gRNA cassette, Cas9 gene, and *NptII* confirmation, respectively. For the identification of insertion/deletions (Indels), 188 bp sequences consisting of *eIF4E* target regions were amplified by primers (Table 5) and cloned into the pTZ57R/T vector, and 20 clones from each line were sent for sequencing.

### 5.5 Phenotypic Screening Against PVY Resistance

The screening of mutated lines for resistance against PVY was carried out in a glass house under controlled environmental conditions. The mutated and wild-type lines were hardened for 2 weeks in pots containing a mixture of sand and peat moss. Triple superphosphate (CaH_2_PO_4_·2H_2_O) at 128 kg per hectare and muriate of potash (KCl) at 485 kg per hectare were applied to strengthen the potato lines. After 2 months of maturation, mutated plants from each line were selected for inoculation of PVY virus by applying viral sap mixed with carborundum powder on the lower side of the leaves. The experiment was conducted in three batches. The control wild-type lines were arranged with six mutated lines and treated with PVY, despite the fact that the control line was PVY susceptible (Hull, 2009). All these lines were screened for PVY resistance using a local virulent PVY strain. The inoculum was prepared to infect the wild-type control and transgenic plants with the virus. The leaf sap of PVY-infected plants was extracted by crushing the leaves in 0.5% sodium acetate solution. The transgenic plants were inoculated with the filtered leaf sap after mixing with carborundum (1 mg/ml) powder. The control lines were inoculated in the same pattern as the mutated lines. PVY from the crude extract was applied to the wild-type plants at various concentrations (1:60 dilutions) to determine the sensitivity of PVY detection. At 7, 15, 30, and 60 dpi, the response of the mutated plants was examined, and data were recorded by observing the mosaic pattern on leaves. Further confirmation was carried out by DAS-ELISA, and the quantitative expression of *eIF4E* in mutated lines was analyzed by RT-qPCR.

### 5.6 ELISA for PVY Confirmation

Screening of PVY was performed by DAS-ELISA in the wild-type and mutated Kruda lines. Leaves from the infected non-transgenic control plants and tolerant/resistant plants mutated for PVY resistance were collected. About 1 g of leaves was ground in distilled water and then the kit-protocol (Catalog# V093) was followed for titer confirmation of PVY in the mutated and control lines. The DAS-ELISA was performed at regular intervals of 7, 15, 30, and 60 dpi. DAS-ELISA was performed for PVS, PLRV, PVA, and PVM with PVY to find out the screening sensitivity of DAS-ELISA.

### 5.7 RNA Extraction, cDNA Synthesis, and Relative Expression

Total RNA was extracted from the inoculated potato leaves using Trizol reagent (Invitrogen, United States) following the manufacturer’s instructions. The extracted RNA was treated with gradient DNase I (Thermo Fisher Scientific, United States) as per the manufacturer’s instructions for the removal of DNA contamination. Complementary DNA (cDNA) was synthesized using the RevertAid First Strand cDNA synthesis kit (Thermo Fisher Scientific, United States). The cDNA was synthesized by using oligo (dT_18_) primers and gene-specific Cas9-reverse primers for *eIF4E* and *Cas9*, respectively. The expression of *eIF4E* upon PVY treatment was analyzed in both mutated and control plants by observing relative expression *via* RT-PCR. The *eIF4E* transcripts were amplified using *St-IF4E* RT-F and *eIF4E* RT-R primers (Table 3), and the functional *Cas9* transcripts were analyzed by using Cas-RT-F and Cas-RT-R primers (Table 3). Reaction mixtures of a volume of 25 µl were prepared using 12.5 µl SYBR Green Real-Time PCR Master Mix (Thermo Fisher Scientific, United States), 0.1 pmole of F&R primers, 2 µl cDNA, and 9.5 µl water. The reaction was performed under optimized conditions using a Bio-Rad iQ5 thermal cycler (Bio-Rad, United States). The *actin-97* gene was used as an endogenous control for expression using an actin-F/actin-R primer set (Table 3). The quantification results were analyzed by the 2^−ΔΔCT^ method (Livak and Schmittgen, 2001). To find the virus copy number, analysis of the absolute expression was performed and each treatment was replicated three times.

#### 5.7.1 Preparation of PVY Templates for Standard Curve Construction for Absolute RT-qPCR

For the quantification of PVY titer in the control and mutated lines, specific stranded templates were generated. The VPg of PVY was amplified by primers (P#4, Table 5) and cloned into a TA-cloning vector. Serial dilutions were prepared to achieve specific nucleic acid quantification values as shown in Table 4, and standard curve was generated by using primers in VPg-RT-F/R (Table 3).

## Data Availability

The original contributions presented in the study are included in the article/Supplementary Material; further inquiries can be directed to the corresponding authors.
