# Corrigendum: CRISPR/Cas9-mediated targeting of susceptibility factor eIF4E-enhanced resistance against Potato virus Y

**DOI:** 10.3389/fgene.2022.1035804

**Published:** 2022-12-05

**Authors:** Azka Noureen, Muhammad Z. Khan, Imran Amin, Tayyaba Zainab, Shahid Mansoor

**Affiliations:** ^1^ Agricultural Biotechnology Division, National Institute for Biotechnology and Genetic Engineering (NIBGE), Pakistan Institute of Engineering and Applied Sciences (PIEAS), Faisalabad, Pakistan; ^2^ University Institute of Biochemistry and Biotechnology (UIBB), Pir Mehr Ali Shah-Arid Agriculture University, Rawalpindi, Pakistan; ^3^ National Centre of Industrial Biotechnology (NCIB), Pir Mehr Ali Shah-Arid Agriculture University, Rawalpindi, Pakistan

**Keywords:** potato viruses, CRISPR-Cas9, eIF4E, PVY, DAS-ELISA, RT-PCR, VPg-EIF4E protein interaction

In the published article, there was an error in Figure 3 as published. We neglected to provide figure labels. The corrected Figure 3 and its caption appears below.

**FIGURE 3:**
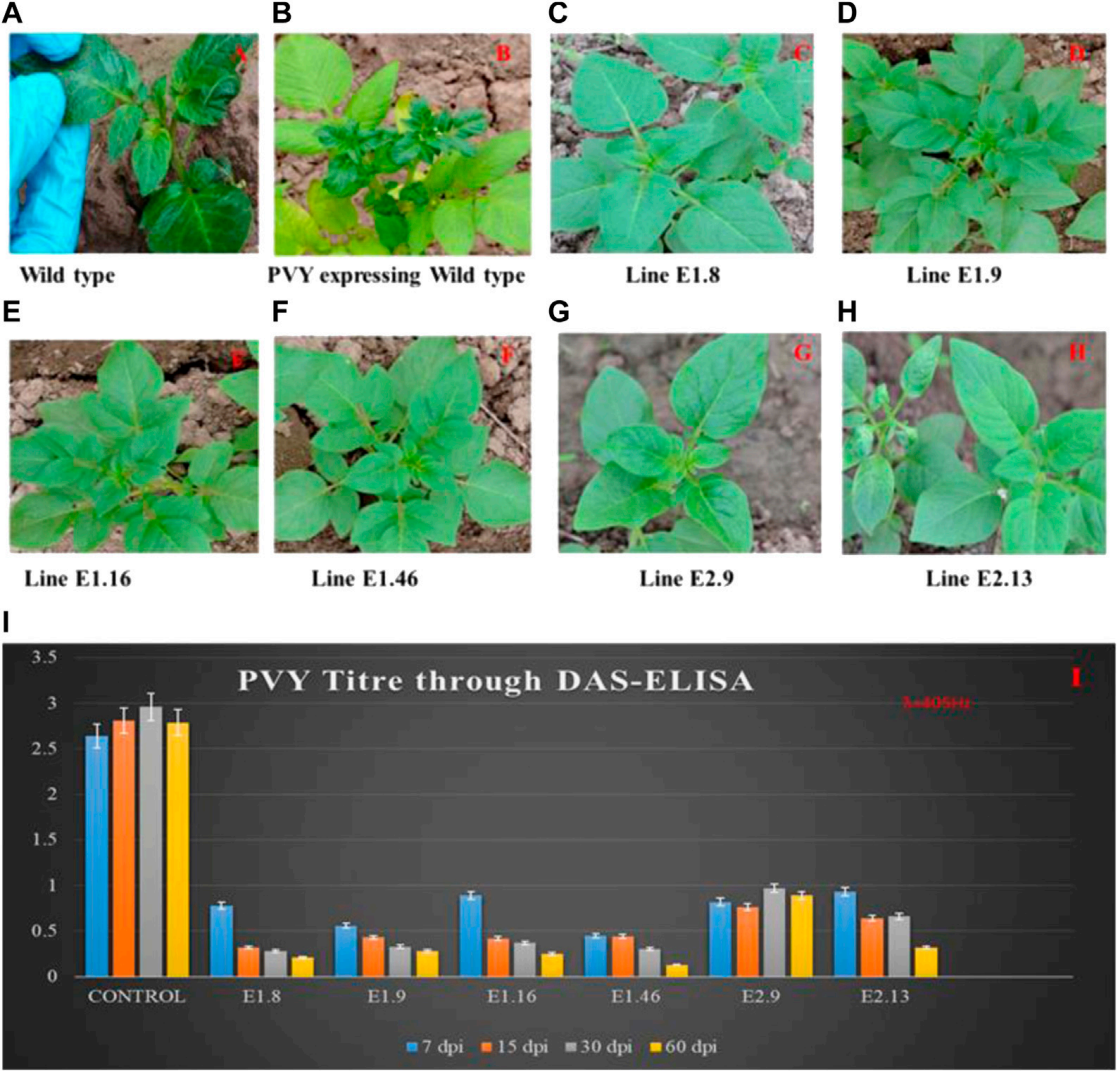
PVY Inoculation and symptoms appearance on control and mutated lines. **(A)** Inoculation of PVY into the control plant; **(B)** virus mosaic pattern appearance on the control plant at 45 dpi; lines **(C)** K_E1.8, **(D)** K_E1.9, **(E)** K_E1.16, and **(F)** K_E1.46 showing strong resistance, and lines **(G)** K_E2.9 and **(H)** K_E2.13 showing tolerance against PVY. **(I)** Determination of PVY titer using DAS-ELISA; The assay was performed to check the PVY titer at regular intervals of 7, 15, 30, and 60 dpi. The control lines showed high titer, while the mutated eIF4E lines K_E1.8, K_E1.9, K_E1.16, K_E1.46, K_E2.9, and K_E2.13 showed virus titer only after 60 days, which was lower than all others.

The authors apologize for this error and state that this does not change the scientific conclusions of the article in any way. The original article has been updated.

