# Supplementary material for: CRISPR/Cas9-Mediated Targeting of Susceptibility Factor eIF4E-Enhanced Resistance Against Potato Virus Y
Source: Front Genet. 2022 Jul 13;13:922019. doi: 10.3389/fgene.2022.922019 (PMC9326172; doi:10.3389/fgene.2022.922019)
Supplement: Supplementary file 1 [file DataSheet1.docx]

**Supplementary Figure 1: Characterization of editing events in tetraploid potato cultivar**


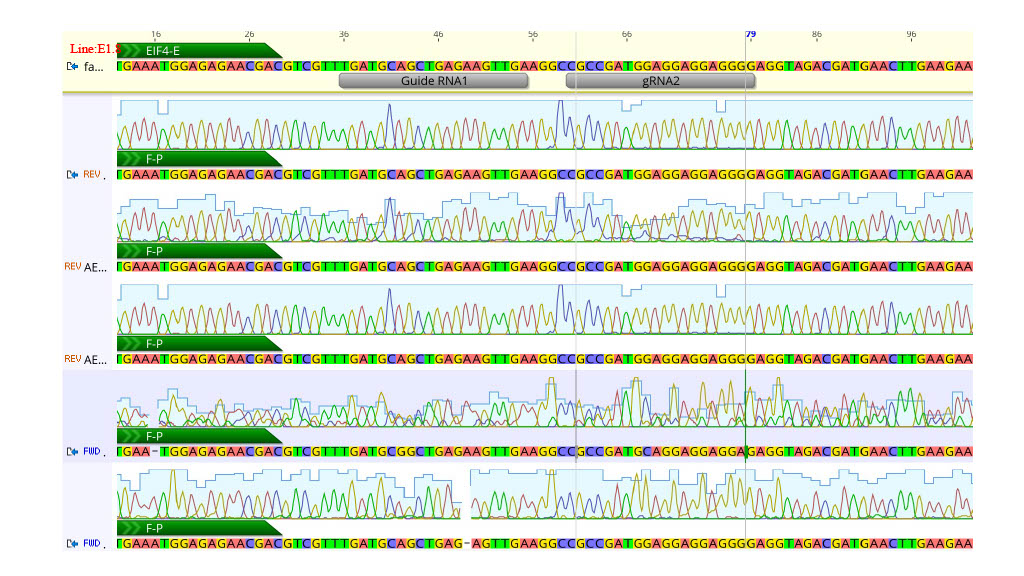


A

A


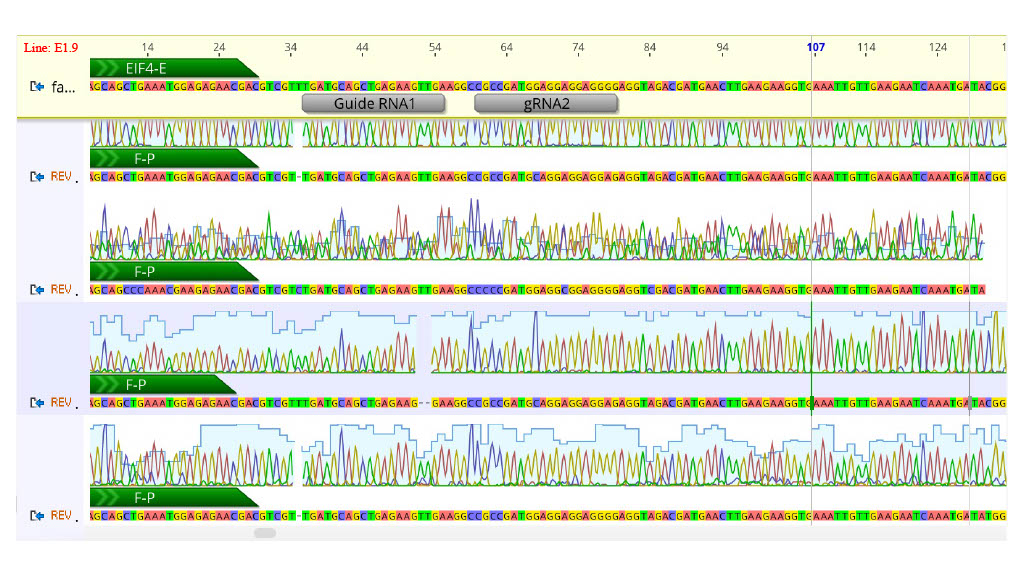


B


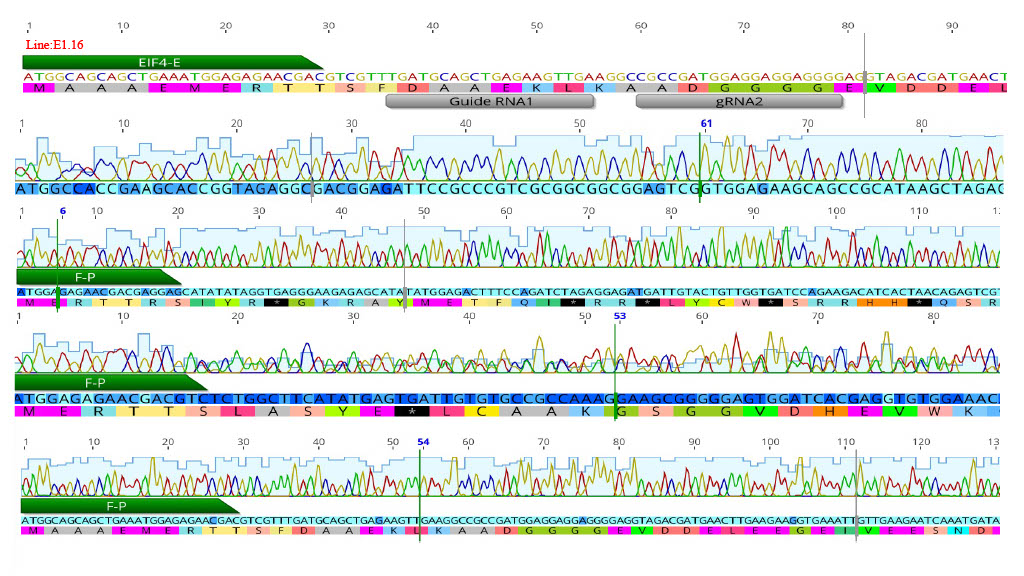


C


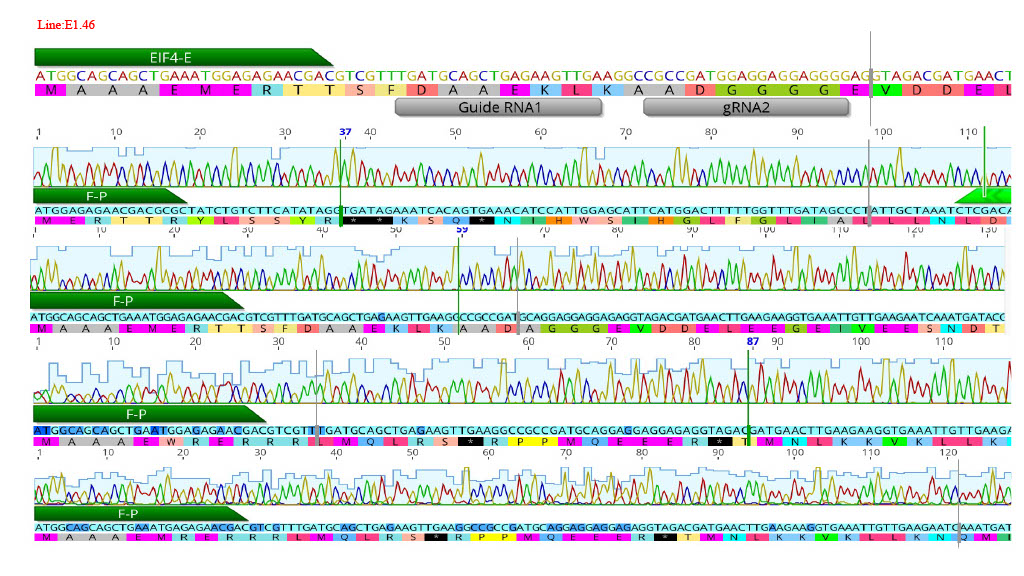


D


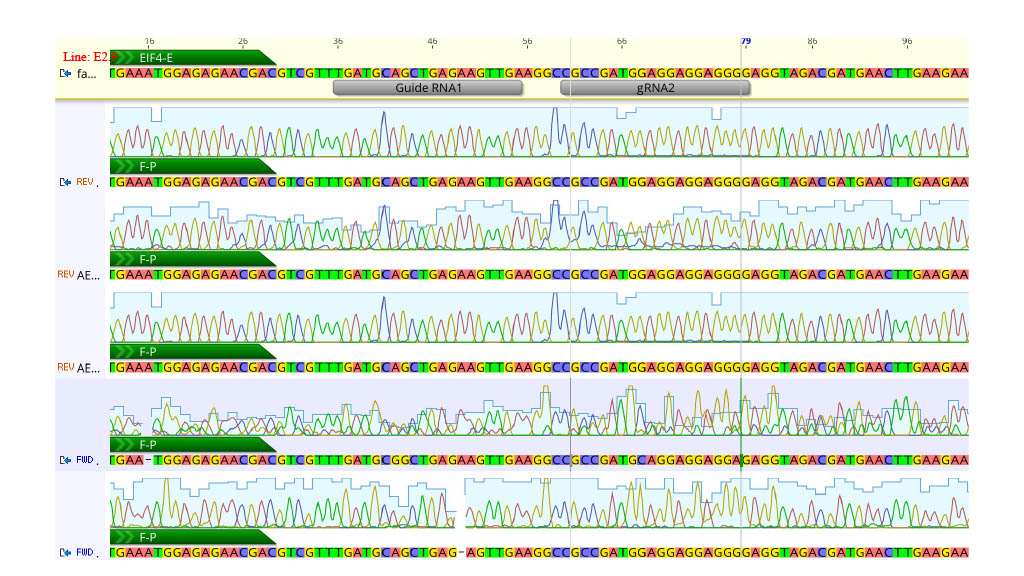


E


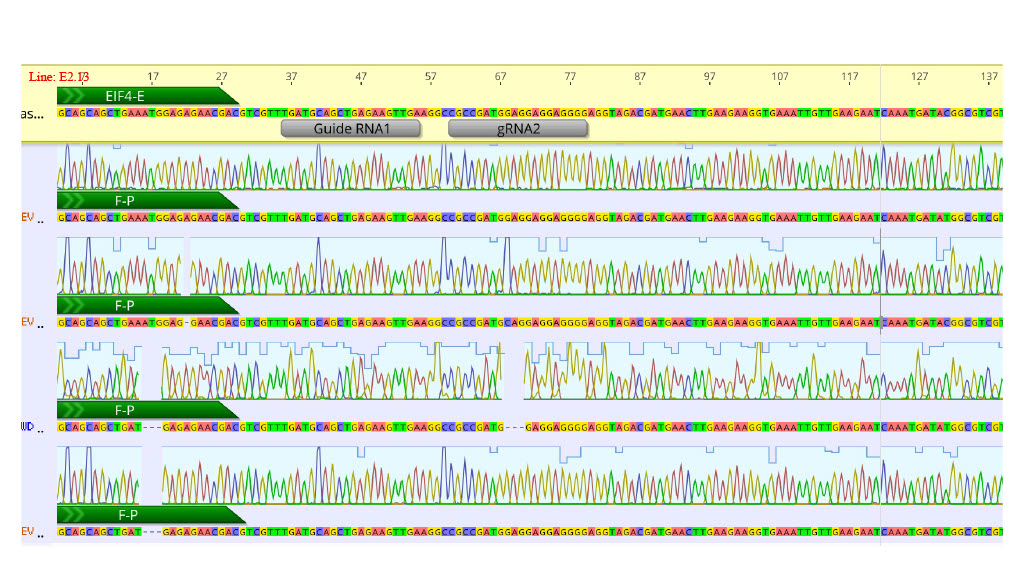


F

**Supplementary Figure 1**: A) Represent the sequence of eIF4E-wild type and edited sequences of line E1.8 with variable edits in all alleles, B) *eIF4E*-wild type and edited sequences of line E1.9 representing editing in tetraploid, C) *eIF4E*-wild type and edited sequences of line E1.16 representing -25bp, -15bp, -25bp deletions and +10bp, +14bp and +35bp insertions D) Exhibiting -139bp, and -1bp deletions and +42bp maximum insertion through NHEJ methods in tetraploid, E) showing the mutations through gRNA2, +1bp, -3bp and -1bp edits and F) -1bp, -2bp, -2b, -3bp and -2bp deletions have been appeared in all possible alleles confirmed through sanger sequencing.

**Supplementary Figure 2: Phenotypic resistant lines in pots and soil**

Supplementary Figure 2: A; Phenotypic assay of edited lines showing the resistance against PVY, B; shifting of PVY-resistant lines from pots to soil for tuber collection.

Supplementary Figure 3: RNA Isolation and standard’s optimization

Supplementary Figure 3: A; is the representation of quality and quantity of RNA-isolation, B; is the representation of amplification and optimization of standards

**Supplementary Figure 4a: Amplifications of whole gene EIF4E-1 gene from 6 non-transgenic Kruda lines**

**Supplementary Figure 4a: Confirmation of gRNA-cassette in p.Chimera vector**

**Supplementary Figure 4b: Confirmation of gRNA-Cassette in PK2GW7-Cas9 vector**

**Supplementary Figure 4c:**

**Supplementary 4a is the representation of uncropped gel picture of wild-type amplification eIF4E-1 gene with +ve and _ve controls. Supplementary figure 4b&4c is the gRNA PCR confirmation with respect to +ve and _ve controls in the p.chimera vector and PK2GW7-Cas9 vector respectively.**
